# Enhanced biohydrogen production from cotton stalk hydrolysate of *Enterobacter cloacae* WL1318 by overexpression of the formate hydrogen lyase activator gene

**DOI:** 10.1186/s13068-020-01733-9

**Published:** 2020-05-22

**Authors:** Qin Zhang, Shaolin You, Yanbin Li, Xiaowei Qu, Hui Jiang

**Affiliations:** 1 College of Biological and Chemical Engineering, Anhui Polytechnic University, Wuhu, 241000 Anhui China; 2 College of Life Science, Tarim University, Alaer, 843300 Xinjiang China

**Keywords:** Biohydrogen production, Formate hydrogen lyase activator gene, Overexpression, Cotton stalk hydrolysate, Recombinant strain, Metabolic flux

## Abstract

**Background:**

Biohydrogen production from lignocellulose has become an important hydrogen production method due to its diversity, renewability, and cheapness. Overexpression of the formate hydrogen lyase activator (*fhlA*) gene is a promising tactic for enhancement of hydrogen production in facultative anaerobic *Enterobacter*. As a species of *Enterobacter*, *Enterobacter cloacae* was reported as a highly efficient hydrogen-producing bacterium. However, little work has been reported in terms of cloning and expressing the *fhlA* gene in *E. cloacae* for lignocellulose-based hydrogen production.

**Results:**

In this study, the formate hydrogen lyase activator (*fhlA*) gene was cloned and overexpressed in *Enterobacter cloacae* WL1318. We found that the recombinant strain significantly enhanced cumulative hydrogen production by 188% following fermentation of cotton stalk hydrolysate for 24 h, and maintained improved production above 30% throughout the fermentation process compared to the wild strain. Accordingly, overexpression of the *fhlA* gene resulted in an enhanced hydrogen production potential (*P*) and maximum hydrogen production rate (*R*_m_), as well as a shortened lag phase time (*λ*) for the recombinant strain. Additionally, the recombinant strain also displayed improved glucose (12%) and xylose (3.4%) consumption and hydrogen yield Y(H_2_/S) (37.0%) compared to the wild strain. Moreover, the metabolites and specific enzyme profiles demonstrated that reduced flux in the competitive branch, including succinic, acetic, and lactic acids, and ethanol generation, coupled with increased flux in the pyruvate node and formate splitting branch, benefited hydrogen synthesis.

**Conclusions:**

The results conclusively prove that overexpression of *fhlA* gene in *E. cloacae* WL1318 can effectively enhance the hydrogen production from cotton stalk hydrolysate, and reduce the metabolic flux in the competitive branch. It is the first attempt to engineer the *fhlA* gene in the hydrogen-producing bacterium *E. cloacae*. This work provides a highly efficient engineered bacterium for biohydrogen production from fermentation of lignocellulosic hydrolysate in the future.

## Background

With the worsening energy crisis and exhaustion of traditional fossil energy dominated by petroleum, energy production based on lignocellulose bioconversion has attracted worldwide attention [1–5]. Hydrogen is the cleanest energy on earth, and has become the most competitively exploited renewable form of energy in the world [6–9]. In recent years, biohydrogen production from lignocellulose has become an important hydrogen production method due to its diversity, renewability, and cheapness [10–13].

In the process of lignocellulose-based hydrogen production via dark fermentation, fermentative microorganisms play an important role, and engineering the alteration of key enzyme genes in the hydrogen generation pathway has become an important way of improving the hydrogen production potential of hydrogen-producing strains [14–16]. Metabolic pathways for microbial hydrogen synthesis vary with microbial species, but can be classified into three types: (1) the mixed-acid fermentation pathway of facultative anaerobes represented by *Enterobacter* (hydrogen production via formate hydrogen lytic reaction); (2) the butyric acid fermentation pathway of obligate anaerobic bacteria represented by *Clostridium* (hydrogen production catalyzed by pyruvate:ferredoxin oxidoreductase); and (3) the NADH regeneration pathway [17–21], the schematic diagram of the three metabolic pathways for fermentative hydrogen production from glucose is represented as Additional file 1: Fig. S1. Amongst these, the mixed acid fermentation pathway, which is mainly catalyzed by the formate hydrogen lyase (FHL), is the most studied and widely used for hydrogen production [22–24]. The FHL complex exists in various microbial genera, nevertheless, it has been particularly studied in *E. coli*, which has been the most extensively characterized at both the physiological and genetic levels [22, 23]. To date, research on the FHL complex in other species of the *Enterobacter* genus is still limited; only a few studies have reported *fhlA* gene expression in *Enterobacter aerogenes* [24, 25], *Enterobacter* sp. CN1 [26] and *Klebsiella* HQ-3 [27], whereas the *fhlA* gene of *Enterobacter cloacae* and its expression have not yet been reported.

In recent years, great efforts have been made in metabolic engineering to improve the hydrogen production potential of hydrogen-producing bacteria. However, most of the engineered hydrogen-producing bacteria reported can only utilize single carbon sources, like glucose, for hydrogen production [28–30]. Studies involving engineered bacteria utilizing lignocellulose and its hydrolysate for hydrogen production are few, as most focus on the simultaneous saccharification and fermentation (SSF) of cellulose and xylose utilization; for example, an l-lactate dehydrogenase gene (*ldh*) deletion in *Caldicellulosiruptor bescii* increased acetate and H_2_ production by 21–34% relative to the wild type [31], and the overexpression of xylulokinase and xylose isomerase in *Klebsiella oxytoca* HP1 increased hydrogen yield by 33.04% and 41.31%, respectively, relative to the wild type [32]. A hydrogen-producing bacterium *Enterobacter cloacae* WL1318, which has been reported to utilize cotton stalk hydrolysate for hydrogen production [33], was obtained by our group. Based on the strain’s intrinsic sugar utilization and hydrogen production properties, the xylose metabolic pathway of *Enterobacter cloacae* WL1318 does not require modification for lignocellulose-based hydrogen production, but requires specific engineering of its biohydrogen synthesis pathway for enhancement of hydrogen production. However, studies regarding this and the cloning and expression of FHL-related genes in *E. cloacae* are currently limited; only studies on [Fe] hydrogenase gene cloning and expression have been reported [34–36].

In this study, in order to understand the regulation of the formate hydrogen lytic pathway for hydrogen production in *E. cloacae* WL1318 and improve its hydrogen production potential using cotton stalk hydrolysate as the fermentative substrate, we cloned an FHL activator gene (*fhlA*) from the wild *E. cloacae* WL1318 to achieve homologous overexpression of *fhlA*. Moreover, the cumulative hydrogen production and dynamics, glucose and xylose consumption, cell growth, and soluble metabolites were analyzed in both the wild type and recombinant *E. cloacae* WL1318.

## Results

### Cloning, analysis and overexpression of the *fhlA* gene in *E. cloacae* WL1318

**Fig. 1:**
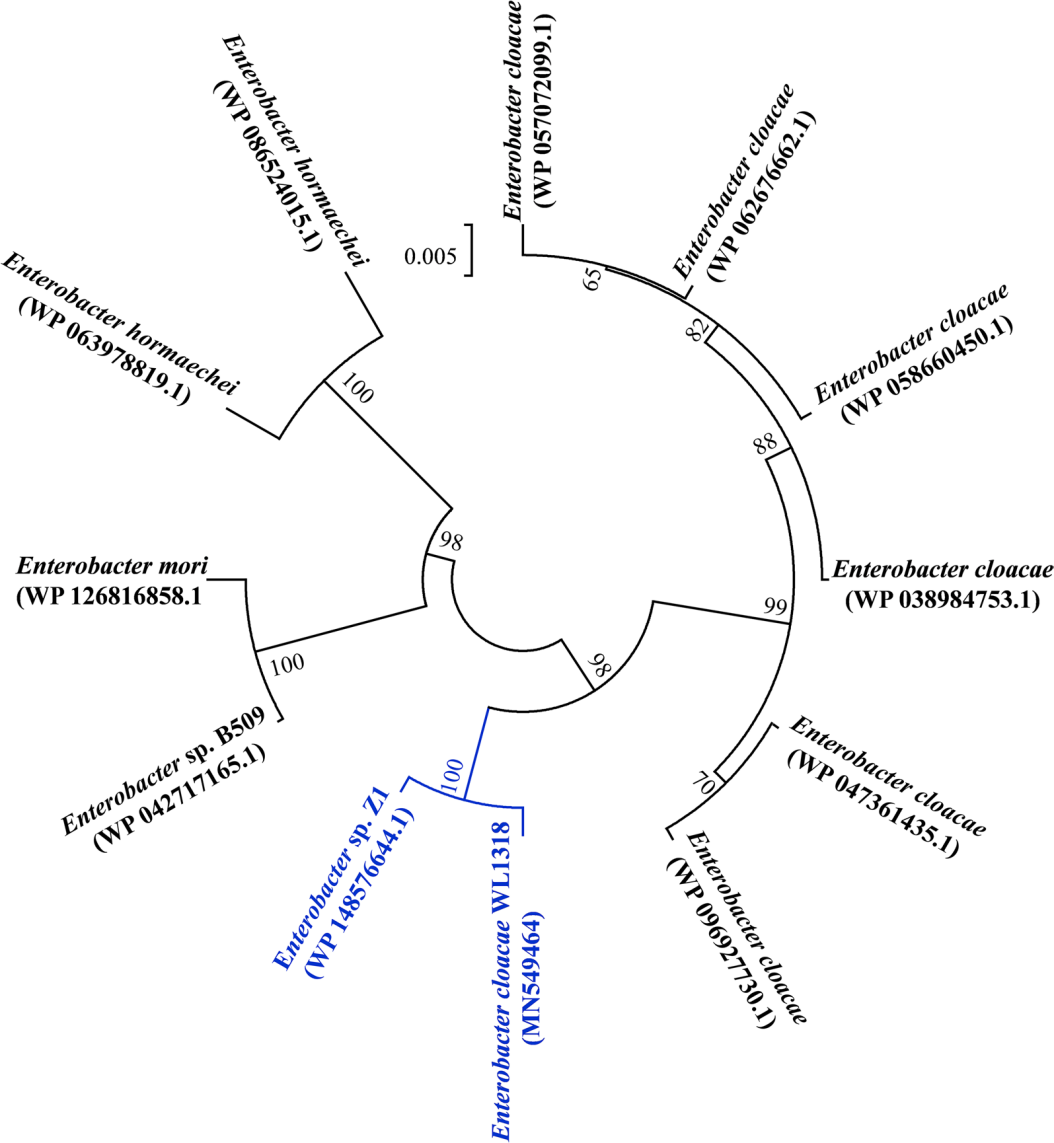
Phylogenetic tree of the formate hydrogen lyase transcriptional activator (FHLA) sequences. The tree was constructed using the neighbor-joining algorithm of the Mega 6.0 program with 1000 bootstrap replicates; the bootstrap values above 60% confidences are displayed. The GenBank accession numbers and strain name are indicated for each protein

The full-length sequence of *fhlA* (2058 bp) was successfully cloned using the primer pair, *fhlA*-fw and *fhlA*-rv, which encoded a peptide of 685 amino acids with a calculated molecular mass of ~ 77 kDa and a predicted pI of 5.78, the nucleotide sequence and amino acid sequence are illustrated in Additional file 1: Figs. S2, S3 separately. The gene sequence has been deposited in the GenBank database under the accession number, MN549464. The results from a BlastP search using the amino acid sequence encoded by *fhlA* to query the GenBank database showed that the peptide was > 95% identity with peptides from different species within the genus, *Enterobacter*. The phylogenetic tree (Fig. 1) showed that the formate hydrogen lyase transcriptional activator (FHLA) protein of *E. cloacae* WL1318 is located in the same clade with *Enterobacter* sp. Z1 (WP_148576644.1), indicating the closest relationship between both proteins. Qiu et al. cloned an *fhlA* gene from *Enterobacter* sp. CN1, whose amino acid sequences showed highest similarity with that of *E. cloacae* subsp. *cloacae* NCTC9493 [26]. Such phenomenon revealed that FHLA may exhibit high similarity among different species of *Enterobacter* genus, rather than limited to the same species. The high identity of the FHLA sequence of *E. cloacae* WL1318 with that of bacteria belonging to *Enterobacter* genus indicated the successful cloning of the full-length *fhlA* gene, which would result in biological activity possessed by the cloned gene, thus the subsequent homologous overexpression of *fhlA* gene might lead to an enhancement of FHL activity and hydrogen production in *E. cloacae* WL1318.

**Fig. 2:**
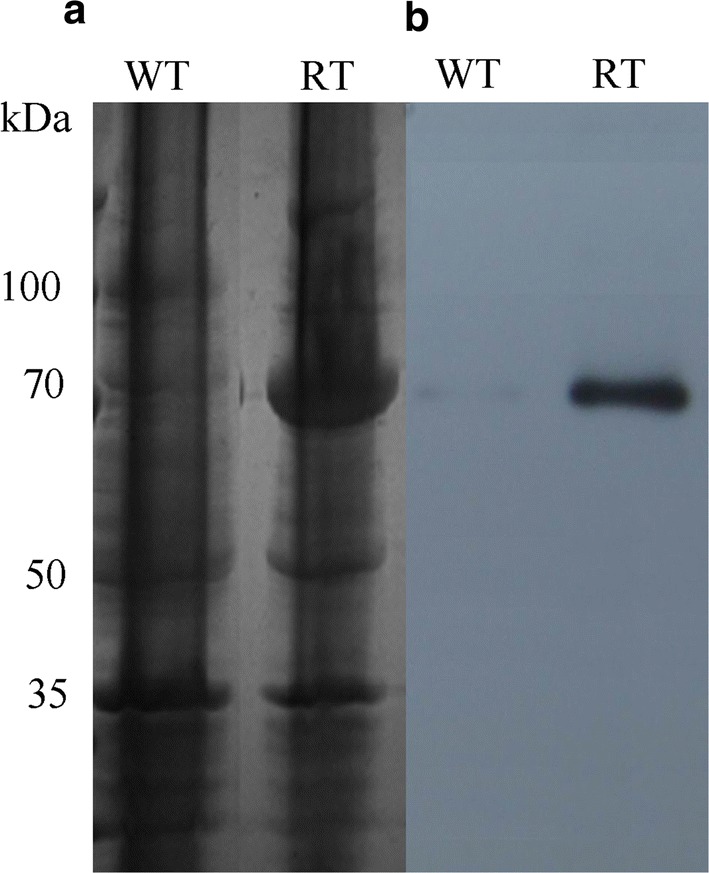
Expression of the *fhlA* gene in *E. cloacae* WL1318. **a** SDS‑PAGE gel was stained with Coomassie Brilliant Blue. **b** Western blot analysis of the expression of the *fhlA*‑encoded protein using anti-6 × His tag antibody. WT: the wild-type strain, RT: the recombinant strain; the numbers denote molecular masses in kDa

In this study, *fhlA* was subcloned using the primer pair, P*fhlA*-fw and P*fhlA*-rv, into pET28a, and this clone was transformed into *E. cloacae* WL1318. The His-FHLA fusion protein expression was evaluated via SDS-PAGE, which showed that the fusion protein had a molecular weight of 77–78 kDa, consistent with the size of the His-tag (0.6 kDa) and the predicted FHLA of 77 kDa (Fig. 2a). The expression of the *fhlA* gene in the recombinant strain was estimated via western blotting, and the level of the encoded protein was observed to be markedly increased (Fig. 2b).

### Enhanced hydrogen production via *fhlA* gene overexpression

**Fig. 3:**
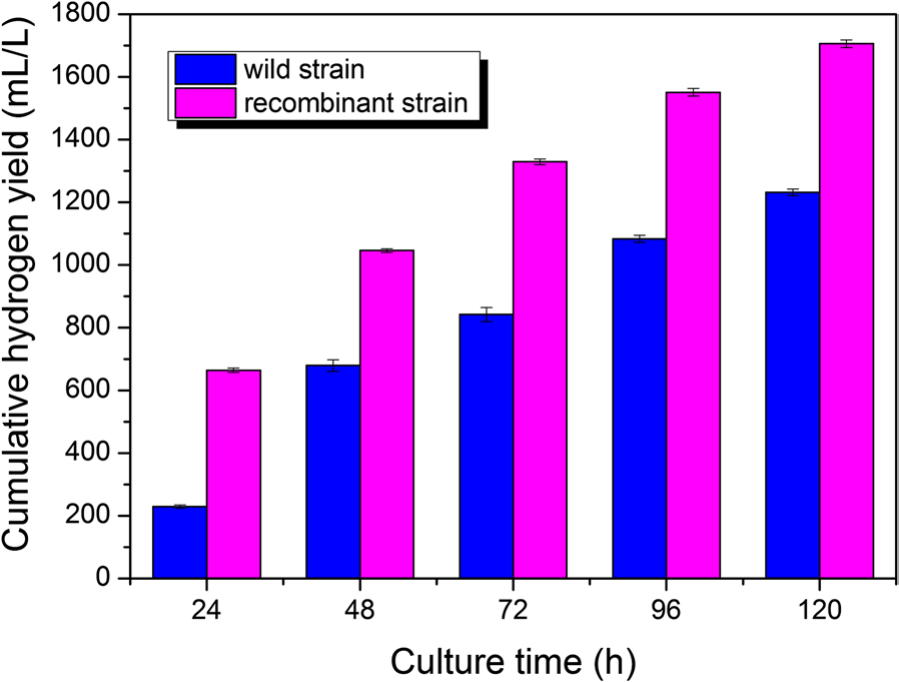
Time course profiles of cumulative hydrogen production for wild and recombinant strains

The time course profiles for cumulative hydrogen production for wild and recombinant strains are shown in Fig. 3. The chart shows that the hydrogen production of the recombinant strain at each fermentation time point was higher than that of the wild strain, and production was particularly enhanced by up to ~ 188% after 24–h fermentation, compared with the wild strain. This indicated that the recombinant strain had a higher hydrogen production potential with *fhlA* overexpression, thereby generating hydrogen gas rapidly and in large quantity during the early fermentation stage (within 24 h). Therefore, the homologous expression of *fhlA* in *E. cloacae* WL1318 significantly enhanced *fhlA* expression in the early fermentation stage. The improvement in cumulative hydrogen production was maintained at > 30% throughout the fermentation period (up to 120 h), indicating that the recombinant strain can also maintain high hydrogen production efficiency during the fermentation process.

Cumulative hydrogen production by the wild and recombinant strains were dynamically fitted by the Gompertz model, and the calculated parameters and correlation coefficients are listed in Table 1. Correlation coefficients with *R*^2^ values higher than 0.98, indicate a good correlation between the experimental data and the model in both the wild and recombinant strains. By comparison, it was discovered that the *P* and *R*_m_ of the recombinant strain were significantly higher than those of the wild strain (Table 1), suggesting that the strain’s *P* and *R*_m_ can be significantly enhanced via *fhlA* overexpression. Additionally, the lag phase time (*λ*) of the recombinant strain was shortened to 2.91 h, which was much shorter than that of the wild type (Table 1), indicating that *fhlA* overexpression shortened the adaptive time and accelerated the fermentative hydrogen production process of the recombinant strain.

**Table 1:** Parameters and correlation coefficients of the Gompertz model dynamically fitted cumulative hydrogen production for wild and recombinant strains

Strains	Dynamically fitted parameters			
	*R*_m_ (mL L^−1^h^−1^)	*P* (mL L^−1^)	*λ* (h)	*R*^2^
Wild strain	12.28	1078.33	12.82	0.9933
Recombinant strain	22.42	1709.00	2.91	0.9826

The main reducing sugars in cotton stalk hydrolysate are glucose and xylose as previously reported [37, 38]. The wild *E. cloacae* WL1318 could utilize both glucose and xylose in cotton stalk hydrolysate, and we found that the insertion of the *fhlA* gene did not change its sugar utilization properties, but enhanced glucose and xylose consumption by 12.0% and 3.4%, respectively (Table 2). Correspondingly, the hydrogen yield Y(H_2_/S) of the recombinant strain increased by 37.0% compared to the wild strain (Table 2). The results indicated that the *fhlA* gene insertion enhanced hydrogen generation, as well as reducing sugar consumption, resulting in an improved hydrogen yield. On the other hand, there was no obvious difference in ΔOD_600_ between the wild and recombinant strains, suggesting that the recombinant strain could maintain a similar growth rate as the wild strain.

**Table 2:** The reducing sugar consumption, increment in OD_600_, bacterial growth efficiency, and hydrogen yield in the wild and recombinant strains

Strains	Reducing sugar consumption		Increment of OD_600_ (ΔOD_600_)	Y(H_2_/S) (mol H_2_/mol sugar)
	Glucose consumption (%)	Xylose consumption (%)		
Wild strain	74.22 ± 0.27	89.01 ± 1.32	2.63 ± 0.01	0.27 ± 0.01
Recombinant strain	83.12 ± 1.21	92.03 ± 0.31	2.67 ± 0.03	0.37 ± 0.01

### Redistribution of metabolic flux influenced by *fhlA* gene overexpression

**Fig. 4:**
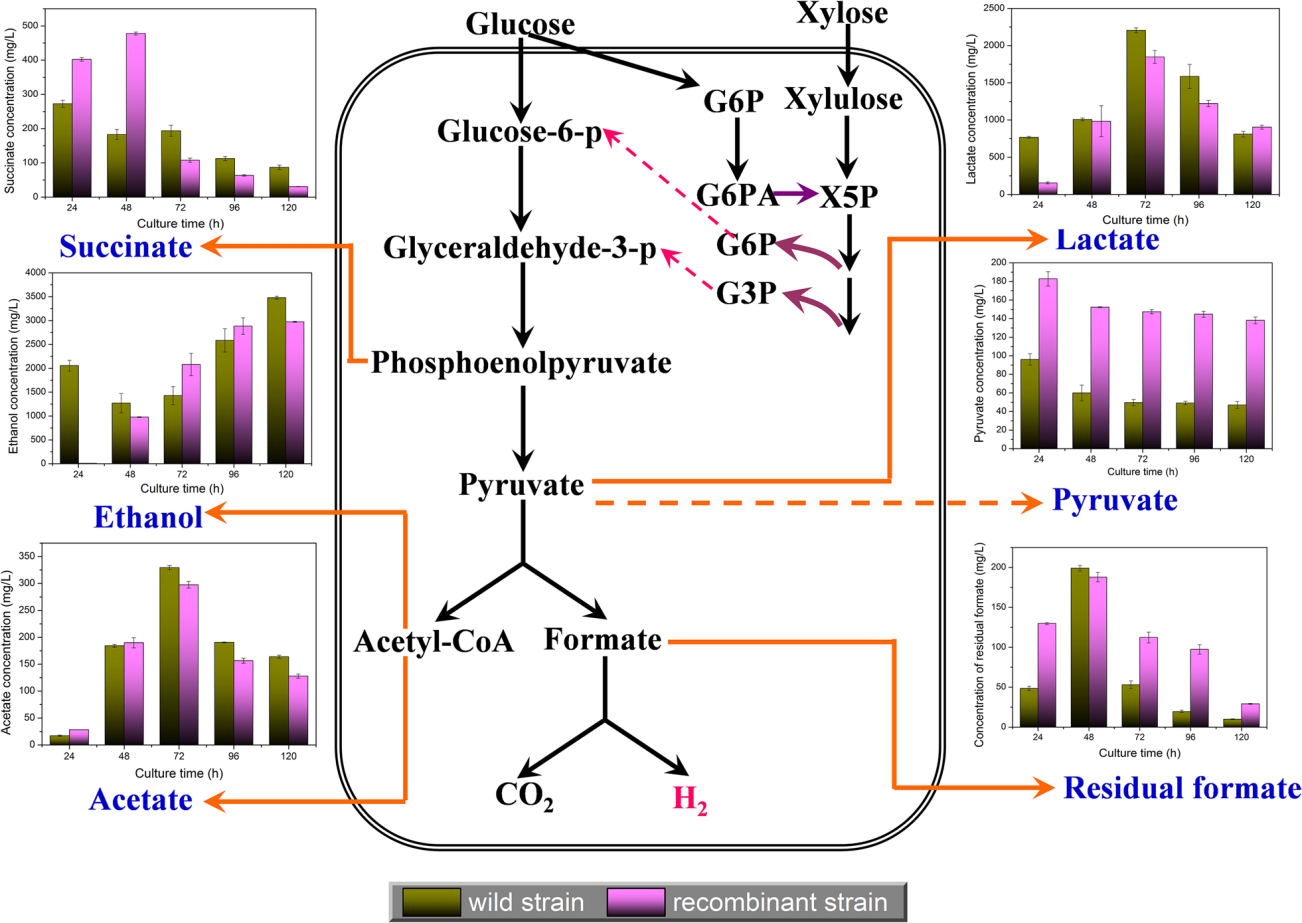
Metabolite profiles of the wild type and recombinant strains reflecting in the hydrogen production metabolic pathway during the entire fermentation stage. G6P: glucose-6-phosphate; G6PA: glucose-6-phosphate acid; X5P: xylulose-5-phosphate; G3P: 3-phosphoglyceric acid

Through metabolic flux analysis based on quasi-steady state, Qu proposed the metabolic pathway of *E. cloacae* WL1318 for hydrogen production from fermentation of cotton stalk hydrolysate as follows: glucose and xylose are metabolized via the Embden–Meyerhof–Parnas (EMP) and Pentose Phosphate (PPP) Pathways, and further converted to pyruvate [39]. Based on the central anaerobic metabolism of *Enterobacter*, pyruvate is split into formate and acetyl-CoA by pyruvate formate lyase; H_2_ and CO_2_ are then produced from formate catalyzed by FHL via the mixed acid fermentation pathway (Fig. 4) [40, 41]. Metabolites, such as lactate, formate, ethanol, acetate, and succinate, are also produced in this pathway. Hence, we measured these metabolites and pyruvate in both the wild and recombinant strains.

After glucose and xylose transporting, most of the carbons were directed to pyruvic acid. Pyruvic acid is the key node compound, which starts the mixed acid fermentation. Significant increases in pyruvic acid during the fermentation stage were detected in the recombinant strain. Compared with the wild strain, pyruvic acid concentration was maintained at > 100 mg/L over a 120-h fermentation period. It may be inferred that the *fhlA* gene insertion increased carbon flux to the pyruvic acid node, which further influenced the flux distribution of the mixed acid fermentation pathway. Succinic acid in the recombinant strain accumulated in the early stage of fermentation with a higher concentration than in the wild strain, but this concentration dropped sharply in the mid-to-late fermentation stage, indicating that succinic acid only competed for carbon flux at the early fermentation stage. Ethanol accumulated at the late fermentation stage in the wild strain, but the concentration decreased slightly in the recombinant strain. Acetic and lactic acid concentrations peaked at the mid-fermentation stage in the recombinant strain, similar to those in the wild strain. Contrastingly, acetic and lactic acid concentrations were lower at most time points during the fermentation stage than in the wild strain, illustrating a reduced flux flow in the competitive branch of hydrogen synthesis in the recombinant strain. Formic acid splitting directly leads to biohydrogen generation in the recombinant strain. Figure 4 shows that the concentrations of residual formic acid were higher during the fermentation stage in the recombinant strain than in the wild strain except at 48 h, indicating the enhancement of carbon flux in the hydrogen generation branch. We think that the increased carbon flux promoted biohydrogen production and maintained more residual formic acid, which might further increase biohydrogen production.

**Fig. 5:**
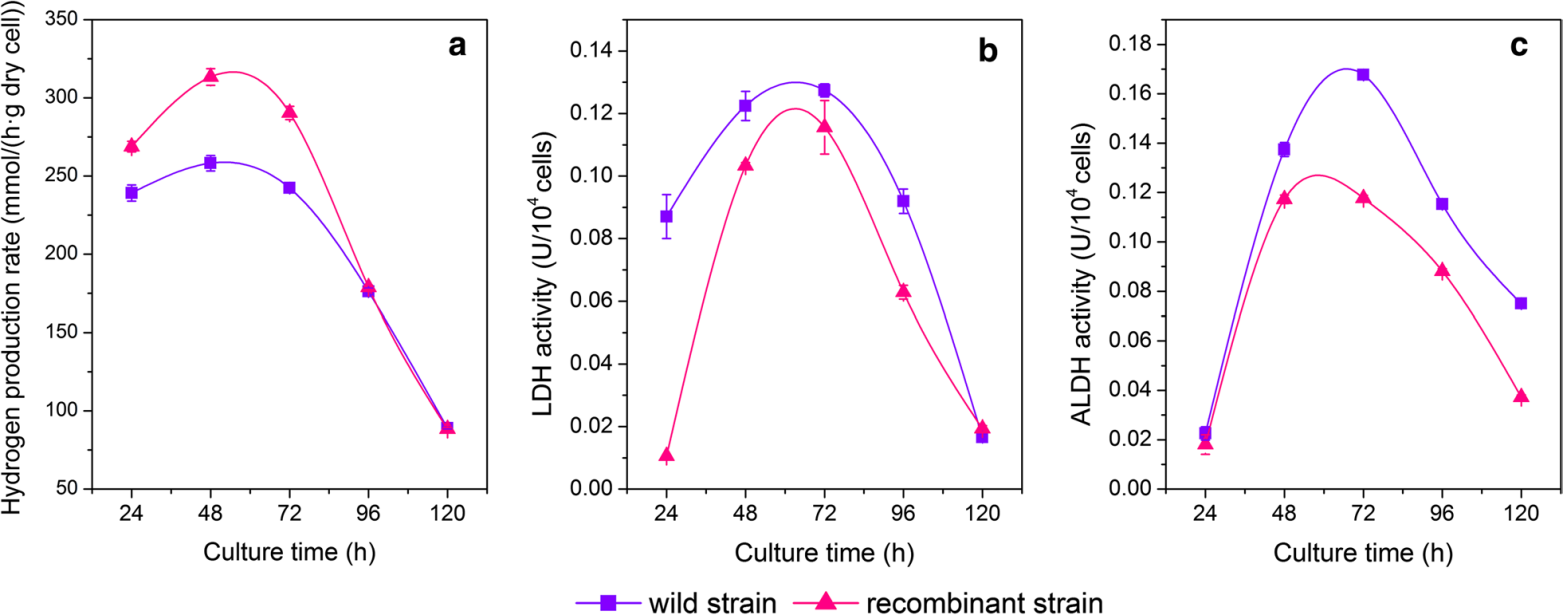
The specific enzyme activity of the wild and recombinant strains

Formate hydrogen lyase (FHL) directly catalyzes the splitting of formic acid into H_2_ and CO_2_. During the hydrogen production process, the FHL activity increased significantly in the recombinant strain from fermenting 24 h to 72 h, and obtained the highest enhancement of about 21% compared to the wild strain (Fig. 5a). This indicated that high FHL activity led to the conversion of formic acid to large amounts to hydrogen gas from the early to the mid stage.

Lactate dehydrogenase (LDH) is a rate-limiting enzyme that catalyzes the conversion of pyruvate to lactic acid, which may decrease the metabolic flux to hydrogen synthesis. The LDH activities varied in a similar trend in the wild and recombinant strains, and both obtained the peak values at the mid-fermentation stage (Fig. 5b), which was also similar to the variation in lactic acid concentration (Fig. 4). Overall, the activities of the recombinant strain were lower than those of the wild strain during the entire fermentation stage (Fig. 5b). Aldehyde dehydrogenase (ALDH) catalyzes the conversion of acetyl-CoA to ethanol and acetic acid and its activities significantly decreased after 48- to 120-h fermentation in the recombinant strain compared to the wild strain (Fig. 5c), indicating that *fhlA* overexpression could decrease the metabolic flux of this branch, which is beneficial for more metabolic flux flow to the biohydrogen synthesis pathway.

## Discussion

FHL is the key enzyme responsible for hydrogen production through formic acid splitting in facultative anaerobic bacteria. The transcription of the FHL complex is mainly controlled by the *fhlA*-encoded protein, FHLA [42, 43]; thus, overexpression of *fhlA* may effectively improve the formate-splitting and hydrogen-producing potential of facultative anaerobes. The method to introduce *fhlA* gene expression has been applied in *E*. *coli* [23], *Enterobacter aerogenes* [24, 25], *Enterobacter* sp. CN1 [26], and *Klebsiella* HQ-3 [27], which has increased hydrogen production to some extent. However, these bacteria only utilized the synthetic medium for hydrogen production, or adding formic acid to promote hydrogen evolution. *E. cloacae* WL1318 has the ability to utilize cotton stalk hydrolysate for hydrogen production. Overexpression of *fhlA* in this strain may cause increased lignocellulose-based hydrogen generation. In the present study, the full-length sequence of *fhlA* was successfully cloned from wild *E. cloacae* WL1318. Using phylogenetic analysis, we discovered that the amino acid sequence of FHLA in *E. cloacae* WL1318 showed a high degree of similarity to that of the bacteria belonging to the genus *Enterobacter*, which would result in functional gene expression with biological activity. Subsequently, *fhlA* in *E. cloacae* WL1318 was homologously overexpressed. To the best of our knowledge, this is the first study to report the expression of *fhlA* in *E. cloacae*. Overexpression of *fhlA* led to enhanced hydrogen production and FHL activity in the recombinant strain compared to the wild *E. cloacae* WL1318. Notably, the increase in cumulative hydrogen production after 24-h fermentation and peak FHL activity after 48-h fermentation was the highest in the recombinant strain, indicating the up-regulation of hydrogen synthesis pathway by *fhlA* overexpression. This finding is consistent with the previous report that *fhlA* overexpression enhances hydrogen production in some *E. coli* strains [22]. In the present study, specific variations in dynamically fitted parameters in the recombinant strain, such as higher *P* and *R*_m_, as well as shorter *λ* than those in the wild strain, reinforced the conclusion that there are positive regulatory effects of *fhlA* overexpression. It is not difficult to explain why the highest enhancement of hydrogen production was obtained in the early fermentation stage (within 24 h), possibly because the shortened *λ* led to a shorter adaptive time and promoted the rapid generation of hydrogen gas in the recombinant strain.

A unique result of this study was that the recombinant strain retained its ability to utilize glucose and xylose in cotton stalk hydrolysate. The overexpression of *fhlA* did not change the intrinsic capability of the strain for co-utilization of glucose and xylose. Instead, the utilization of these sugars was improved in the recombinant strain due to the overexpression of the gene. This result makes the *E. cloacae* WL1318-*fhlA* a promising strain for enhanced lignocellulose-based hydrogen production. In recent years, introducing gene engineered strains into the lignocellulose-based hydrogen production chain would be promising for hydrogen energy manufacturing. However, most of the previously reported recombinant strains require the modification of their sugar utilization pathways to effectively metabolize the reducing sugar in lignocellulosic hydrolysate. For example, when a recombinant *Klebsiella oxytoca* HP1 was modified using xylulokinase and xylose isomerase gene overexpression, it thus obtained the ability to utilize bamboo stalk hydrolysate for hydrogen production [32]. The recombinant *E. cloacae* WL1318-*fhlA* obtained in the current study required no such modification of the sugar utilizing pathway. Therefore, once it is implemented in lignocellulose-based hydrogen production, the process will become considerably simpler and more efficient than traditional techniques.

This study improves the understanding of the metabolic flux alteration of key node pathways that are influenced by *fhlA* overexpression. In this study, we measured the key node metabolites typically present in the mixed-acid fermentation pathway, as reported previously [22, 24, 25, 40, 41]. Nevertheless, most of the previous studies solely measured the end metabolites and simply compared the variations between the recombinant and wild-type strains. Our measurement of the alteration of key node metabolites in the hydrogen production pathways during the entire fermentation stage was distinct. Through the current dynamic measurement, we discovered that the carbon flux directed to the pyruvic acid node was significantly enhanced in the recombinant strain. Pyruvate is the most important intermediate metabolite in central metabolic pathway. The pyruvate node not only combines the EMP and PPP pathways, but also connects to the TCA cycle and initiates some important fermentation pathways. The enhanced carbon flux in the pyruvate node may improve the occurrence of downstream events. In the competitive branches of hydrogen synthesis, the ethanol production branch exhibited high ethanol concentration and shared similar variation trends in both the recombinant and wild strains; their metabolism favors ethanol production to maintain the balance of reducing equivalents inside the cell [44]. In the succinate, acetic, and lactic acid production branches, the recombinant strain showed decreased carbon flux, compared to that of the wild strain. In contrast, in the hydrogen synthesis pathway, the residual formic acid concentration in the recombinant strain increased by 112–398%, compared to the wild strain since 72 h fermentation, we inferred that the recombinant strain accumulated sufficient formic acid to generate hydrogen gas. In general, the reduced flux flow in the competitive branch of hydrogen synthesis and formic acid accumulation in the mid-to-late fermentation stages in the recombinant strain strengthened the positive regulation of *fhlA* overexpression on hydrogen production. However, such regulation is limited to the compound level and is not involved at the gene expression level; therefore, further transcriptome analyses need to be performed to elucidate the variations of key gene expression in the hydrogen production network.

## Conclusions

Overexpression of *fhlA* gene is a promising tactic for enhancing hydrogen production in facultative anaerobic *Enterobacter*. In this study, *fhlA* gene was effectively cloned and overexpressed in *E. cloacae* WL1318. Compared to the wild strain, the recombinant strain significantly enhanced hydrogen production from the fermentation of cotton hydrolysate for 24 h by ~ 188%, and maintained > 30% improved production throughout the fermentation stage. Additionally, the recombinant strain also displayed enhanced *P* and *R*_m_, shortened lag-phase time, and improved reducing sugar consumption and hydrogen yield Y(H_2_/S) compared to the wild strain. Moreover, the metabolite profile demonstrated that flux reduction in the competitive branch, including succinic, acetic, and lactic acids, and ethanol generation, and increases in flux in the pyruvate node and formate splitting branch benefited hydrogen synthesis. These trends were confirmed by activity assays of the enzymes, FHL, LDH, and ALDH.

## Methods

### Strains and plasmids

Strains and plasmids used in this study are provided in Table 3. *E. cloacae* WL1318, a bacterium reported to utilize cotton stalk hydrolysate for hydrogen production [33], served as the wild-type strain from which the recombinant strain was constructed. A previously described [33] growth medium was used for the wild strain: glucose 10 g/L, xylose 10 g/L, beef extract 5 g/L, peptone 10 g/L, NaCl 5 g/L, KH_2_PO_4_ 0.5 g/L, and MgSO_4_·7H_2_O 0.5 g/L. *E. coli* DH5α served as the host strain for plasmid construction. Recombinant strain *E. cloacae* WL1318-*fhlA* and *E. coli* DH5α were grown in LB medium and the antibiotics, 50 μg/mL ampicillin and 10 μg/mL kanamycin, were prepared and added into the media when required.

**Table 3:** Strains and plasmids used in this study

Strain or plasmid	Genotype and relevant characteristics	Reference or source
*E. coli* DH5α	F-, φ 80dlacZ ΔM15, Δ (lacZYA -argF) U169, deoR, recA1, endA1, hsdR17 (rK^−^, mK^+^), phoA, supE44, λ^−^, thi-1, gyrA96, relA1	Sangon
*E. cloacae* WL1318	Wild type	[26]
*E. cloacae* WL1318-*fhlA*	*E. cloacae* WL1318 containing pET28a-*fhlA*	This study
pUCm-T	TA Cloning vector, Amp^r^	Sangon
pET28a	Prokaryotic expression vector, Kan^r^	Miaolingbio
pET28a-*fhlA*	*fhlA* in EcoRI–XhoI sites of pET28a	This study

### Preparation of cotton stalk hydrolysate

Cotton stalks were harvested from a cotton field in Xinjiang Alaer, China, dried, milled and sifted to 20-mesh size before being hydrolyzed. The fermentable sugar solution of the cotton stalk hydrolysate was prepared following optimum hydrolysis, then detoxified and decolorized as previously described [37, 38, 45]. The main sugar components in the hydrolysate solution were glucose and xylose [38, 46], which were modified to a previously determined optimum concentration (total reducing sugar concentration was 40 g/L) and used as the substrate medium in the following fermentation experiments.

### Cloning of the *fhlA* gene in *Enterobacter cloacae* WL1318 and sequence analysis

The total DNA of *E. cloacae* WL1318 was extracted using an Ezup Column Bacterial Genomic DNA Purification Kit (Sangon, Shanghai, China). Strains and plasmids used in this study are listed in Table 3. The sequence of the *E. cloacae* WL1318 *fhlA* gene was obtained via PCR (polymerase chain reaction) using the primer pair, *fhlA*-fw and *fhlA*-rv (Table 4), which was designed based on the open reading frames (ORF) of FHLA proteins from related facultative anaerobic bacteria (the amino acid sequences were illustrated in Additional file 1: Fig. S4). Related protein sequences were retrieved using the BLAST program in the NCBI database, multiple sequence alignments of amino acid sequences were performed using Clustal W program (http://www.clustal.org), and a phylogenetic tree was constructed via the MEGA 6.0 software using the neighbor-joining algorithm with a bootstrap support value of 1000 replicates.

**Table 4:** Primers designed for cloning and overexpression of the *fhlA* gene

Primers	Sequence	Source
*fhlA*-fw	ATGAGCGATCTTGGACAGCAG	This study
*fhlA*-rv	TTAATTCAGGCTCTCTTTATCA	This study
P*fhlA*-fw	ATGAATTCGAGCGATCTTGGACAGCAG	This study
P*fhlA*-rv	CGGCTCGAGTTAATTCAGGCTCTCTTTATCA	This study

### Sub-cloning and overexpression of the *fhlA* gene in *Enterobacter cloacae* WL1318

The gene *fhlA* was purified, and sub-cloned via PCR amplification using the primers, P*fhlA*-fw and P*fhlA*-rv, containing EcoRI and XhoI sites (underlined), respectively. The PCR product was purified and ligated to the pUCm-T vector, the purified plasmid, pUCm-T-*fhlA*, was digested with EcoRI and XhoI, and inserted into the multiple cloning site of the prokaryotic expression vector, pET28a, forming the recombinant plasmid, pET28a-*fhlA* (Kan^r^). The recombinant plasmid with the *fhlA* insert was purified and confirmed by double enzyme digestion and sequencing, then transformed into the *E. cloacae* WL1318 competent cells by electroporation (2500 V, 200 Ω, 25 μF) to obtain the recombinant strain *E. cloacae* WL1318-*fhlA*.

Cells of the recombinant strain were induced with 1 mmol/L IPTG for 6 h, harvested, and lysed using an ultrasonic cell disruptor (3 s, 1 s, 15 min) (Scientz-IID, SCIENTZ, Ningbo, China). Proteins were collected by centrifugation, and analyzed via 12% sodium dodecyl sulfate polyacrylamide gel electrophoresis (SDS-PAGE) to primarily confirm FHLA protein expression. Western blots were performed via the Novel One Step Western Blot Kit I (Sangon, Shanghai, China), anti-6 × His-tag antibody (Sangon, Shanghai, China) was used as the primary antibody at dilution 1/1500, the specific *fhlA* encoded protein was finally examined using the W-TMB chromogenic kit (Sangon, Shanghai, China).

### Batch fermentation for biohydrogen production

Single colonies of the recombinant strain *E. cloacae* WL1318-*fhlA* were selected and inoculated in 3 mL LB medium (including 10 μg/mL kanamycin) and cultured overnight at 37 °C, 180 r/min, then 3-mL broth was inoculated with 30 mL fresh seed medium (glucose 10 g/L, xylose 10 g/L, beef extract 5 g/L, NaCl 5 g/L, peptone 10 g/L, KH_2_PO_4_ 0.5 g/L, MgSO_4_·7H_2_O 0.5 g/L, and 10 μg/mL kanamycin), which was then shake-cultivated at 37 °C and 180 r/min until the optical density value at 600 nm (OD_600_) reached 0.4–0.6 (after 2–3 h). Next, sterilized IPTG was added to a final concentration of 1 mmol/L to induce expression at 37 °C for 6 h. The entire 30 mL seed broth was introduced into the fermentation medium composed of the following: cotton stalk hydrolysate (reducing sugar concentration 40 g/L) 1000 mL, beef extract 5 g/L, peptone 10 g/L, NaCl 5 g/L, KH_2_PO_4_ 0.5 g/L, MgSO_4_·7H_2_O 0.5 g/L, kanamycin 10 μg/mL, and IPTG at a final concentration of 1 mmol/L. The fermenter was well sealed to maintain anaerobic conditions for hydrogen production and cultivation was performed at 37 °C for 120 h. The fermentation method for hydrogen production was also applied to the wild strain without the addition of kanamycin and IPTG. All the experiments were carried out in triplicates. The volume of the fermentative hydrogen was examined thrice every 24 h and summed to obtain the daily hydrogen production, which was measured at each fermentation time point, was accumulated and calculated as the cumulative hydrogen production in the corresponding fermentation period. The concentrations of the main soluble metabolites, glucose, and xylose in the cotton stalk hydrolysate were measured at 24-h intervals.

### Analytical methods and calculations

The hydrogen gas volume was measured via 1 mol/L NaOH displacement in an inverted burette, and the gas concentration was examined using a hand-held hydrogen detector (KP810H20, Henan Zhong’an Electronic Detection Technology Co Ltd, Zhengzhou, China). At each sampling time, bacterial growth was measured via the OD_600_ using a UV–visible spectrophotometer (7230G, Jinghua Instruments Co. Ltd., Shanghai, China). The rest of the aqueous samples were centrifuged at 8000×*g* for 10 min and filtered through syringe filters with 0.22 μm membranes before analysis. The total concentration of reducing sugars in the broth was determined via the 3,5-dinitryl-salicylic acid reagent (DNS) method [47]. Glucose concentration was measured using a glucose detection kit (Comin Biotechnology, Suzhou, China) via the principle of glucose oxidation and colorimetry, using a UV–visible spectrophotometer (7230G, Jinghua Instruments Co Ltd, Shanghai, China). Xylose concentration was measured using a xylose detection kit (ZZStandard, Shanghai, China) via the xylose dehydrogenation reaction and detection of NADH formation using a UV–visible spectrophotometer (7230G, Jinghua Instruments Co. Ltd., Shanghai, China). The concentrations of soluble metabolites, such as succinate, lactate, acetate, pyruvate, formate and ethanol were measured using detection kits (ZZStandard, Shanghai, China) according to the manufacturers’ instructions.

The reducing sugar (glucose or xylose) consumption (%, w/w) in the cotton stalk hydrolysate was calculated as a percentage of the initial sugar concentration in the fermentative medium. The difference between the OD_600_ value in the final fermentation broth (120 h) and that in the initial fermentation broth (0 h) was described as the increment in OD_600_ (ΔOD_600_). The hydrogen yield Y(H_2_/S) (mol H_2_/mol sugar) was defined as hydrogen concentration generated from the consumed reducing sugar in the cotton stalk hydrolysate.

The kinetic parameters of the cumulative hydrogen production in batch fermentation experiments were calculated using the modified Gompertz model (Eq. 1) [48] as follows:

1 $$H = P \times \exp \left\{ { - \exp \left[ {\frac{{R_{\text{m}} \times e}}{P}(\lambda - t) + 1} \right]} \right\},$$

where *H* is the cumulative hydrogen production (mL/L), *P* is the hydrogen potential (mL/L), *R*_m_ is the maximum hydrogen production rate (mL/(L h)), *e* is 2.71828, *λ* is the lag phase time (h), and *t* is the culture time (h). The kinetic parameters (*P*, *R*_m_, and *λ*) were estimated via Sigmaplot software 12.

### Assay of FHL activity and specific related metabolic enzyme activity

FHL activity was assayed as described by Yoshida et al. [22]. The specific hydrogen production rate was measured as the rate of hydrogen produced at 37 °C from a stirred cell suspension at an OD_600_ of 1.0 in 50 mL PBS in the presence of 100 mmol/L sodium formate. The volumetric hydrogen production rate was measured at 37 °C by injecting 25 mmol/L formic acid into 100 mL cell suspension in a mixing reactor. The hydrogen concentration was examined using a hand-held hydrogen detector (KP810H20; Henan Zhong’an Electronic Detection Technology Co Ltd, Zhengzhou, China).

LDH activity was examined using an LDH kit (Comin, Suzhou, China) based on the principle of lactic acid oxidation and colorimetry. ALDH activity was determined using an ALDH kit (Comin, Suzhou, China) based on the principle of aldehyde oxidation and colorimetry.

## Supplementary information

**Additional file 1: Fig. S1.** Metabolic pathways for fermentative hydrogen production from glucose. **Fig. S2.** Nucleotide sequence of the formate hydrogen lyase activator (*fhlA*) gene of *E. cloacae* WL1318; **Fig. S3.** Amino acid sequence of the formate hydrogen lyase activator (FHLA) of *E. cloacae* WL1318; **Fig. S4.** Reference amino acid sequences of FHLA in related species of *Enterobacter* for construction of the phylogenetic tree.

## Data Availability

All data generated or analyzed during this study are included in this published article and its additional files.

## Abbreviations

FHL: Formate hydrogen lyase
FHLA: Formate hydrogen lyase activator
ORF: Open reading frame
SDS-PAGE: Sodium dodecyl sulfate polyacrylamide gel electrophoresis
EMP: Embden–Meyerhof–Parnas pathway
PPP: Pentose phosphate pathway
G6P: Glucose-6-phosphate
G6PA: Glucose-6-phosphate acid
X5P: Xylulose 5-phosphate
G3P: 3-Phosphoglyceric acid
LDH: Lactate dehydrogenase
ALDH: Aldehyde dehydrogenase
PBS: Phosphate buffered solution
DNS: 3,5-Dinitryl-salicylic acid reagent
