# Supplementary material for: Enhanced biohydrogen production from cotton stalk hydrolysate of Enterobacter cloacae WL1318 by overexpression of the formate hydrogen lyase activator gene
Source: Biotechnol Biofuels. 2020 May 22;13:94. doi: 10.1186/s13068-020-01733-9 (PMC7245044; doi:10.1186/s13068-020-01733-9)
Supplement: Supplementary file 1 — Additional file 1: Fig. S1. Metabolic pathways for fermentative hydrogen production from glucose. Fig. S2. Nucleotide sequence of the formate hydrogen lyase activator (fhlA) gene of E. cloacae WL1318; Fig. S3. Amino acid sequence of the formate hydrogen lyase activator (FHLA) of E. cloacae WL1318; Fig. S4. Reference amino acid sequences of FHLA in related species of Enterobacter for construction of the phylogenetic tree. [file 13068_2020_1733_MOESM1_ESM.docx]

**Additional file 1**

**Enhanced biohydrogen production from cotton stalk hydrolysate of *Enterobacter cloacae* WL1318 by overexpression of the formate hydrogen lyase activator gene**

Qin Zhang^1^[[1]](#footnote-1)^^, Shaolin You^1^, Yanbin Li^1^, Xiaowei Qu^2^, Hui Jiang^2^

^1^College of Biological and Chemical engineering, Anhui Polytechnic University, Wuhu, Anhui, 241000, China; ^2^College of Life Science, Tarim University, Alaer, Xinjiang, 843300, China

*** Corresponding author

Prof Qin Zhang

Email: jhtabszq@163.com; zhangqin@ahpu.edu.cn


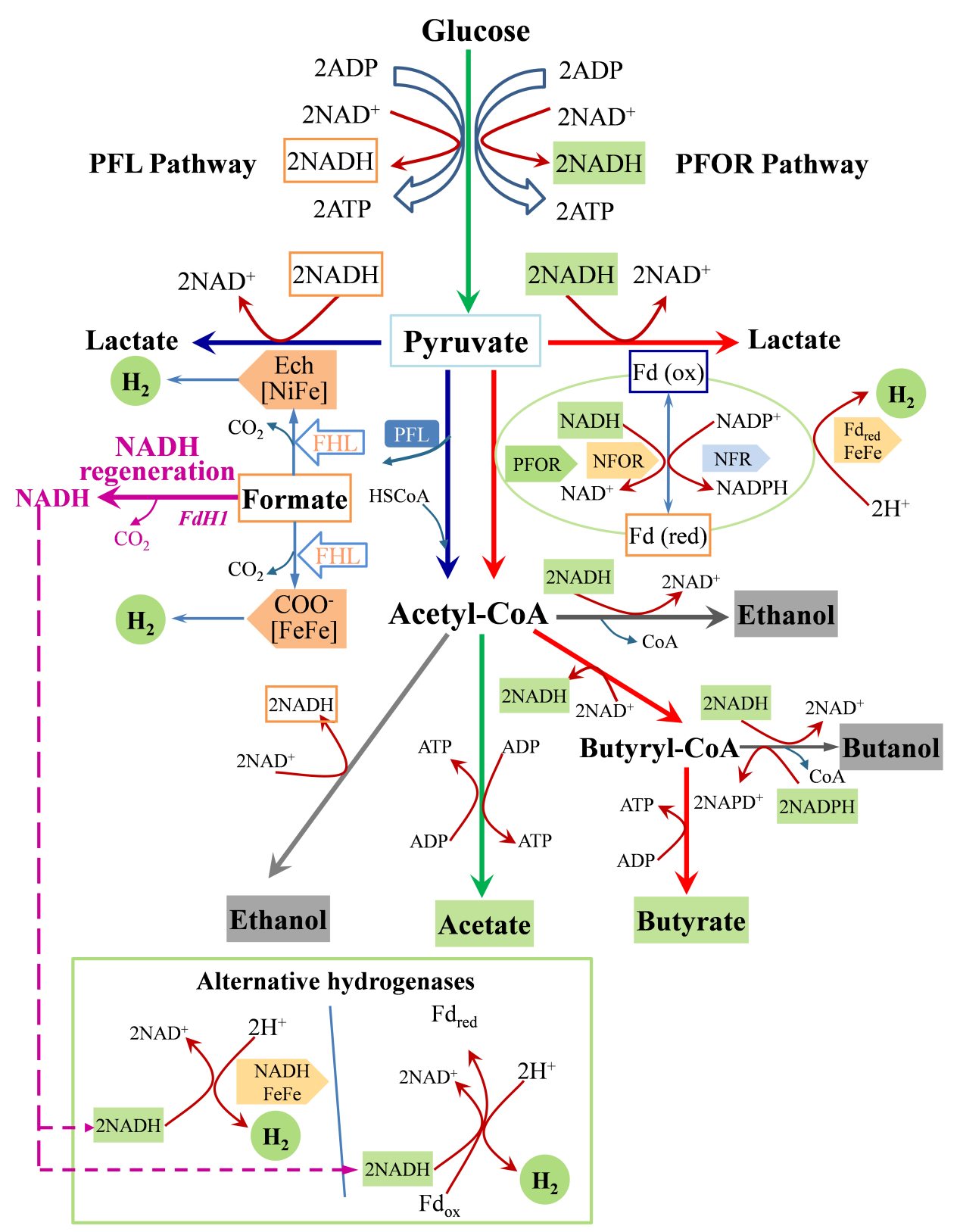


**Fig. S1. Metabolic pathways for fermentative hydrogen production from glucose. The main schematic diagram was quoted from Mohanraj et al (2019) [19], the purple marked part was quoted from Lu et al (2010) [20].**

ATGAGCGATCTTGGACAGCAGGGCCTGTTTGACATTACGCGCACACTTTTACAGCAGCCCGATCTTGGCGCGCTGAGCGATGCCCTGACGCGGCTGGTCAGGCAATCAGCACTGGCAGACAGCGCCGCAATTGTGCTCTGGCATAGCGCAAGCCATCGCGCGAGCTACTATTCAACGCGCGACAATGGCAAAGCGTTTGAGTACGAGGATGAAACCTACCTCGCTCATGGTCCTGTCCGGCGTATTCTTTCCCGCCCGGAGGCGCTGCACTGCAATTTTGAGGAATTCCGCGAGGCGTGGCCGATGCTGGCGCAGAGTCAGTTATACCCGCCTTTCGGCCATTACAGCCTGCTGCCACTGGCGGTGGAAGGGCATATCTTCGGCGGCTGCGAGTTTATTCGTCACACCGACCAGCCGTGGAGCGAGGCGGAATACGAGCGTCTGCACACCTTCACCCAGATTGTGGCCGTGGTCGCGGAGCAAATTCAAAGCCGCGTCACCAATAATGTGGATTACGACCTGCTGAGCCGCGAACGTGACAATTTCCGGATCCTGGTGGCCATCACCAACGCGGTGCTTTCGCGCCTCGATATGGATGAGCTGGTCAGCGAAGTCTCAAAAGAGATCCACCACTATTTCAAAATCGACGCCATCAGTATTGCGCTCCGGGGGCACCGGAAAGGCAAGCTGAACATCTACTCCACCCACTATCTTGATGAGGCAAACCCCGCCCACGAACAGAGTGAAGTGGATGAGGCGGGCACGCTCTCTGAGCGGGTATTTAAAAGCAAAGAGATCCTGCTGCTCAATCTCAACGAGCAGGATGCGCTCGCGCCTTATGAGCGGATGCTGTTTAACACCTGGGGGAATAAAATCCAGA

CGCTGTGCCTGCTGCCGCTGATGTCCGGCAATACTATGCTTGGTGTCCTGAAGCTGGCGCAGTGTGAAGAGGGCGTCTTTACCACCGCCAACCTGAAACTGTTGCGCCAGATTGCCGAGCGTATCTCCATCGCACTGGATAACGCGCTGGCCTATCAGGAGATCCACCGTCTGAAAGAACGGCTGGTGGATGAAAACCTGGCGCTGACGGAACAGCTCAACAACGTGGACAGCGAGTTTGGCGAAATCATCGGCCGCAGCGATGCCATGTACAGCGTGCTGAAACAGGTGGAGATGGTGGCGCAAAGTAACAGTACGGTGCTGATCCTCGGTGAAACCGGCACCGGTAAAGAGCTGATTGCCCGTGCGATCCACAACCTCAGCAACCGTAACAGCCGCCGCATGGTGAAGATGAACTGCGCCGCTATGCCCGCAGGCCTGCTGGAAAGCGATCTCTTTGGCCACGAACGCGGCGCGTTTACCGGTGCCAGCAGCCAGCGTCTGGGCCGTTTCGAGCTGGCGGACAAAAGCTCGTTGTTCCTTGATGAAGTGGGCGACATGCCTCTGGAGCTGCAGCCCAAACTGCTGCGCGTCCTGCAGGAGCAGGAGTTTGAACGTCTTGGCAGTAACAAACTTATCCAGACTGACGTGCGGCTCATTGCCGCCACCAACCGCGATCTGAAAAAAATGGTCGCCGACCGCGAGTTTCGAAGCGACCTCTATTATCGCCTGAACGTCTTCCCAATCTGCCTGCCGCCCCTGCGTGAACGGCCGGAAGATATCCCTCTGCTGGTCAAAGCGTTTACCGCCAAAATAGCCCGCCGGATGGGCCGCAACATCGACAGTATCCCCGCCGAGACGCTACGTACCCTCTCCTCGATGGAGTGGCCGGGTAACGTCCGTGAACTGGAAAACGTCATTGAACGTGCGGTATTGCTGACGCGCGGTAACGTACTGCAACTCTCCCTGCCCGAAGTTTCCCTGCCCGAAACCCCGGTTACAGCCACCGACGTGGCGCAGGAAGGTGAAGACGAATATCAGCTGATCATGCGCGTGCTGAAAGAGACTAACGGTGTCGTGGCCGGACCGAAAGGTGCCGCCCAGCGACTGGGGCTAAAACGCACCACGCTGCCCTCGCGCATGAAACGTCTCGGGATTGATAAAGAGAGCCTGAATTAA

**Fig. S2. Nucleotide sequence of the formate hydrogen lyase activator ( *fhlA*) gene of *E. cloacae* WL1318**

MSDLGQQGLFDITRTLLQQPDLGALSDALTRLVRQSALADSAAIVLWHSASHRASYYSTRDNGKAFEYEDETYLAHGPVRRILSRPEALHCNFEEFREAWPMLAQSQLYPPFGHYSLLPLAVEGHIFGGCEFIRHTDQPWSEAEYERLHTFTQIVAVVAEQIQSRVTNNVDYDLLSRERDNFRILVAITNAVLSRLDMDELVSEVSKEIHHYFKIDAISIALRGHRKGKLNIYSTHYLDEANPAHEQSEVDEAGTLSERVFKSKEILLLNLNEQDALAPYERMLFNTWGNKIQTLCLLPLMSGNTMLGVLKLAQCEEGVFTTANLKLLRQIAERISIALDNALAYQEIHRLKERLVDENLALTEQLNNVDSEFGEIIGRSDAMYSVLKQVEMVAQSNSTVLILGETGTGKELIARAIHNLSNRNSRRMVKMNCAAMPAGLLESDLFGHERGAFTGASSQRLGRFELADKSSLFLDEVGDMPLELQPKLLRVLQEQEFERLGSNKLIQTDVRLIAATNRDLKKMVADREFRSDLYYRLNVFPICLPPLRERPEDIPLLVKAFTAKIARRMGRNIDSIPAETLRTLSSMEWPGNVRELENVIERAVLLTRGNVLQLSLPEVSLPETPVTATDVAQEGEDEYQLIMRVLKETNGVVAGPKGAAQRLGLKRTTLPSRMKRLGIDKESLN

**Fig. S3. Amino acid sequence of the formate hydrogen lyase activator (FHLA) of *E. cloacae* WL1318**

>WP_096927730.1 formate hydrogenlyase transcriptional activator [*Enterobacter cloacae*]

MPYTPMSDLGQQGLFDITRTLLQQPDLGALSDALTRLVRQSALADSAAIVLWHSGSHRASYFSTRENGKAFEYEDETYLAHGPIRRILSRPEALHCNFEEFRQAWPMLARSALYQPFGHYSLLPLAVEGHIFGGCEFIRNTDQPWSEAEYERLHTFTQIVAVVAEQIQSRVSNNVDYDLLSRERDNFRILVAITNAVLSRLDMDELVSEVSKEIHHYFKIDAISIALRGHRKGKLNIYSTHYLDEANPAHEQSEVDEAGTLSERVFKSKEILLLNLSEQDVMAPYERMLFNIWGNKIQTLCLLPLMSGNTMLGVLKLAQCEEGVFTTANLKLLRQIAERISIALDNALAYQEIHRLKERLVDENLALTEQLNNVDSEFGEIIGRSDAMYSVLKQVEMVAQSDSTVLILGETGTGKELIARAIHNLSNRNSRRMVKMNCAAMPAGLLESDLFGHERGAFTGASSQRLGRFELADKSSLFLDEVGDMPLELQPKLLRVLQEQEFERLGSNKLIQTDVRLIAATNRDLKKMVADREFRSDLYYRLNVFPICLPPLRERPEDIPLLVKAFTAKIARRMGRNIDSIPAETLRTLSSMEWPGNVRELENVIERAVLLTRGNVLQLSLPEVSLAETPVTATDVAQEGEDEYQLIVRVLKETNGVVAGPKGAAQRLGLKRTTLLSRMKRLGIDKESLI

>WP_047361435.1 formate hydrogenlyase transcriptional activator [*Enterobacter cloacae*]

MPYTPMSDLGQQGLFDITRTLLQQPDLGALSDALTRLVRQSALADSAAIVLWHSGSHRASYFSTRENGKAFEYEDETYLAHGPIRRILSRPEALHCNFEEFRQAWPMLARSALYQPFGHYSLLPLAVEGHIFGGCEFIRNTDQPWSEAEYERLHTFTQIVAVVAEQIQSRVSNNVDYDLLSRERDNFRILVAITNAVLSRLDMDELVSEVSKEIHHYFKIDAISIALRGHRKGKLNIYSTHYLDEANPAHEQSEVDEAGTLSERVFKSKEILLLNLSEQDVMAPYERMLFNIWGNKIQTLCLLPLMSGNTMLGVLKLAQCEEGVFTTANLKLLRQIAERISIALDNALAYQEIHRLKERLVDENLALTEQLNNVDSEFGEIIGRSDAMYSVLKQVEMVAQSDSTVLILGETGTGKELIARAIHNLSNRNSRRMVKMNCAAMPAGLLESDLFGHERGAFTGASSQRLGRFELADKSSLFLDEVGDMPLELQPKLLRVLQEQEFERLGSNKLIQTDVRLIAATNRDLKKMVADREFRSDLYYRLNVFPICLPPLRERPEDIPLLVKAFTAKIARRMGRNIDSIPAETLRTLSSMEWPGNVRELENVIERAVLLTRGNVLQLSLPEVSLAETPVTATDVAQDGEDEYQLIVRVLKETNGVVAGPKGAAQRLGLKRTTLLSRMKRLGIDKESLI

>WP_038984753.1 formate hydrogenlyase transcriptional activator [*Enterobacter cloacae*]

MPYTPMSDLGQQGLFDITRTLLQQPDLGALSDALTRLVRQSALADSAAIVLWHSGSHRASYFSTRENGKAFEYEDETYLAHGPIRRILSRPEALHCNFEEFRQAWPMLARSALYQPFGHYSLLPLAVEGHIFGGCEFIRNTDQPWSEAEYERLHTFTQIVAVVAEQIQSRVSNNVDYDLLSRERDNFRILVAITNAVLSRLDMDELVSEVSKEIHHYFKIDAISIALRGHRKGKLNIYSTHYLDEANPAHEQSEVDEAGTLSERVFKSKEILLLNLSEQDVMAPYERMLFNIWGNKIQTLCLLPLMSGNTMLGVLKLAQCEEGVFTTANLKLLRQIAERISIALDNALAYQEIHRLKERLVDENLALTEQLNNVDSEFGEIIGRSDAMYSVLKQVEMVAQSDSTVLILGETGTGKELIARAIHNLSNRNSRRMVKMNCAAMPAGLLESDLFGHERGAFTGASSQRLGRFELADKSSLFLDEVGDMPLELQPKLLRVLQEQEFERLGSNKLIQTDVRLIAATNRDLKKMVADREFRSDLYYRLNVFPICLPPLRERPEDIPLLVKAFTAKIARRMGRNIDSIPAETLRTLSSMEWPGNVRELENVIERAVLLTRGNVLQLSLPEVSLPESPATATDVAQDGEDEYQLIVRVLKETNGVVAGPKGAAQRLGLKRTTLLSRMKRLGIDKESLI

>WP_058660450.1 formate hydrogenlyase transcriptional activator [*Enterobacter cloacae*]

MPYTPMSDLGQQGLFDITRTLLQQPDLGALSDALTRLVRQSALADSAAIVLWHSGSHRASYFSTRENGKAFEYEDETYLAHGPIRRILSRPEALHCNFEEFRQAWPMLARSALYQPFGHYSLLPLAVEGHIFGGCEFIRNTDQPWSEAEYERLHTFTQIVAVVAEQIQSRVSNNVDYDLLSRERDNFRILVAITNAVLSRLDMDELVSEVSKEIHHYFKIDAISIALRGHRKGKLNIYSTHYLDEANPAHEQSEVDEAGTLSERVFKSKEILLLNLSEQDVMAPYERMLFNIWGNKIQTLCLLPLMSGNTMLGVLKLAQCEEGVFTTANLKLLRQIAERISIALDNALAYQEIHRLKERLVDENLALTEQLNNVDSEFGEIIGRSDAMYSVLKQVEMVAQSDSTVLILGETGTGKELIARAIHNLSNRNSRRMVKMNCAAMPAGLLESDLFGHERGAFTGASSQRLGRFELADKSSLFLDEVGDMPLELQPKLLRVLQEQEFERLGSNKLIQTDVRLIAATNRDLKKMVADREFRSDLYYRLNVFPICLPPLRERPEDIPLLVKAFTAKIARRMGRNIDSIPAETLRTLSSMEWPGNVRELENVIERAVLLTRGNVLQLSLPEVSLPDSPTAATDVAQDGEDEYQLIVRVLKETNGVVAGPKGAAQRLGLKRTTLLSRMKRLGIDKESLI

>WP_062676662.1 formate hydrogenlyase transcriptional activator [*Enterobacter cloacae*]

MPYTPMSDLGQQGLFDITRTLLQQPDLGALSDALTRLVRQSALADSAAIVLWHSGSHRASYFSTRENGKAFEYEDETYLAHGPIRRILSRPEALHCNFEEFRQAWPMLARSALYQPFGHYSLLPLAVEGHIFGGCEFIRNTDQPWSEAEYERLHTFTQIVAVVAEQIQSRVSNNVDYDLLSRERDNFRILVAITNAVLSRLDMDELVSEVSKEIHHYFKIDAISIALRGHRKGKLNIYSTHYLDEANPAHEQSEVDEAGTLSERVFKSKEILLLNLSEQDVMAPYERMLFNIWGNKIQTLCLLPLMSGNTMLGVLKLAQCEEGVFTTANLKLLRQIAERISIALDNALAYQEIHRLKERLVDENLALTEQLNNVDSEFGEIIGRSDAMYSVLKQVEMVAQSNSTVLILGETGTGKELIARAIHNLSNRNSRRMVKMNCAAMPAGLLESDLFGHERGAFTGASSQRLGRFELADKSSLFLDEVGDMPLELQPKLLRVLQEQEFERLGSNKLIQTDVRLIAATNRDLKKMVADREFRSDLYYRLNVFPICLPPLRERPEDIPLLVKAFTAKIARRMGRNIDSIPAETLRTLSSMEWPGNVRELENVIERAVLLTRGNVLQLSLPEVSLPDSPAAATDVAQDGEDEYQLIVRVLKETNGVVAGPKGAAQRLGLKRTTLLSRMKRLGIDKESLI

>WP_057072099.1 formate hydrogenlyase transcriptional activator [*Enterobacter cloacae*]

MPYTPMSDLGQQGLFDITRTLLQQPDLGALSDALTRLVRQSALADSAAIVLWHSGSHRASYFSTRENGKAFEYEDETYLAHGPIRRILSRPEALHCNFEEFRQAWPMLARSALYQPFGHYSLLPLAVEGHIFGGCEFIRNTDQPWSEAEYERLHTFTQIVAVVAEQIQSRISNNVDYDLLSRERDNFRILVAITNAVLSRLDMDELVSEVSKEIHHYFKIDAISIALRGHRKGKLNIYSTHYLDEANPAHEQSEVDEAGTLSERVFKSKEILLLNLSEQDVMAPYERMLFNIWGNKIQTLCLLPLMSGNTMLGVLKLAQCEEGVFTTANLKLLRQIAERISIALDNALAYQEIHRLKERLVDENLALTEQLNNVDSEFGEIIGRSDAMYSVLKQVEMVAQSDSTVLILGETGTGKELIARAIHNLSNRNSRRMVKMNCAAMPAGLLESDLFGHERGAFTGASSQRLGRFELADKSSLFLDEVGDMPLELQPKLLRVLQEQEFERLGSNKLIQTDVRLIAATNRDLKKMVADREFRSDLYYRLNVFPICLPPLRERPEDIPLLVKAFTAKIARRMGRNIDSIPAETLRTLSSMEWPGNVRELENVIEHAVLLTRGNVLQLSLPEVSLPDSPAAATDVAQDGEDEYQLIVRVLKETNGVVAGPKGAAQRLGLKRTTLLSRMKRLGIDKESLI

>WP_086524015.1 formate hydrogenlyase transcriptional activator [*Enterobacter hormaechei]*

MPYTPMSDLGQQGLFDITRTLLQQPDLGALSDALTRLVRQSALADSAAIVLWHSATHRASYFSTRDNGKIFEYEDETFLAHGPVRRILSRPEALHCNFDQFRQAWPKLAESNLYHPFGHYSMLPLAVEGQIFGGCEFIRDTDQPWSEAEYERLHTFTQIVAVVAEQIQSRVTNNVDYDLLSRERDNFRILVAITNAVLSRLDMDELVSEVSKEIHHYFKIDAISIALRGNRKGKLNIYSTHYLDEANPAHEQSEVDEAGTLSERVFKSKEILLLNLNEQDPVAPYERMLFNTWGNKIQTLCLLPLMSGNTMLGVLKLAQCDEAVFTTANLKLLRQIAERISIALDNALAYQEIHRLKERLVDENLALTEQLNNVDSEFGEIIGRSDAMYSVLKQVEMVAQSDSTVLILGETGTGKELIARAIHNLSNRNSRRMVKMNCAAMPAGLLESDLFGHERGAFTGASSQRLGRFELADKSSLFLDEVGDMPLELQPKLLRVLQEQEFERLGSNKLIQTDVRLIAATNRDLKKMVADREFRSDLYYRLNVFPICLPPLRERPEDIPLLVKAFTAKIARRMGRNIDSIPAETLRTLSAMEWPGNVRELENVIERAVLLTRGNVLQLSLPEVTLSETTVTATELAKEGEDEYQLIMRVLKETNGVVAGSKGAAQRLGLKRTTLLSRMKRLGIDKESLN

>WP_063978819.1 formate hydrogenlyase transcriptional activator [*Enterobacter hormaechei*]

MPYTPMSDLGQQGLFDITRTLLQQPDLGALSDALTRLVRQSALADSAAIVLWHSATHRASYFSTRDNGKIFEYEDETFLAHGPVRRILSRPEALHCNFDQFRQAWPKLAESNLYHPFGHYSMLPLAVEGQIFGGCEFIRDTDQPWSEAEYERLHTFTQIVAIVAEQIQSRVTNNVDYDLLSRERDNFRILVAITNAVLSRLDMDELVSEVSKEIHHYFKIDAISIALRGNRKGKLNIYSTHYLDEANPAHEQSEVDEAGTLSERVFKSKEILLLNLNEQDPVAPYERMLFNTWGNKIQTLCLLPLMSGNTMLGVLKLAQCDEAVFTTANLKLLRQIAERISIALDNALAYQEIHRLKERLVDENLALTEQLNNVDSEFGEIIGRSDAMYSVLKQVEMVAQSDSTVLILGETGTGKELIARAIHNLSNRNSRRMVKMNCAAMPAGLLESDLFGHERGAFTGASSQRLGRFELADKSSLFLDEVGDMPLELQPKLLRVLQEQEFERLGSNKLIQTDVRLIAATNRDLKKMVADREFRSDLYYRLNVFPICLPPLRERPEDIPLLVKAFTAKIARRMGRNIDSIPAETLRTLSAMEWPGNVRELENVIERAVLLTRGNVLQLSLPEVSLAETTVAATEVAKDGEDEYQLILRVLRETNGVVAGPKGAAQRLGLKRTTLLSRMKRLGIDKESLN

>WP_126816858.1 formate hydrogenlyase transcriptional activator [*Enterobacter mori*]

MPYTPMSDLGQQGLFDITRTLLQQPDLGALSDALTRLVRQSALADSAAIVLWHSGTHRASYYSTRDNGKAFEYEDETYLAHGPVRRILSRPEVLHCNFAEFRMAWPRLAESNLYQPFGHYCMLPLAAEGQIFGGCEFIRTTDQPWSEAEYERLHTFTQIVAVVAEQIQSRVTNNVDYDLLSRERDNFRILVAITNAVLSRLDMDELVSEVSKEIHHYFKIDAISIALRGHRKGKLNIYSTHYLDEANPAHEQSEVDEAGTLSERVFKSKEILLLNLNEQDPVAPYERMLFNTWGNKIQTLCLLPLMSGNTMLGVLKLAQCEEGVFTTANLKLLRQIAERISIALDNALAYQEIHRLKERLVDENLALTEQLNNVDSEFGEIIGRSDAMYSVLKQVEMVAQSDSTVLILGETGTGKELIARAIHNLSNRNSRRMVKMNCAAMPAGLLESDLFGHERGAFTGASSQRLGRFELADKSSLFLDEVGDMPLELQPKLLRVLQEQEFERLGSNKLIQTDVRLIAATNRDLKKMVTDREFRSDLYYRLNVFPICLPPLRERPEDIPLLVKAFTAKIARRMGRNIDSIPAETLRTLSSMEWPGNVRELENVIERAVLLTRGSVLQLSLPEISLPDETLVAAEVAQNGEDEYQLIMRVLKETNGVVAGPKGAAQRLGLKRTTLLSRMKRLGIDKESLV

>WP_042717165.1 formate hydrogenlyase transcriptional activator [*Enterobacter* sp. B509]

MPYTPMSDLGQQGLFDITRTLLQQPDLGALSDALTRLVRQSALADSAAIVLWHSGTHRASYYSTRDNGKAFEYEDETYLAHGPVRRILSRPEVLHCNFAEFRMAWPKLAESNLYQPFGHYCMLPLAAEGQIFGGCEFIRTTDQPWSEAEYERLHTFTQIVAVVAEQIQSRVTNNVDYDLLSRERDNFRILVAITNAVLSRLDMDELVSEVSKEIHHYFKIDAISIALRGHRKGKLNIYSTHYLDEANPAHEQSEVDEAGTLSERVFKSKEILLLNLNEQDPVAPYERMLFNTWGNKIQTLCLLPLMSGNTMLGVLKLAQCEEGVFTTANLKLLRQIAERISIALDNALAYQEIHRLKERLVDENLALTEQLNNVDSEFGEIIGRSDAMYSVLKQVEMVAQSDSTVLILGETGTGKELIARAIHNLSNRNSRRMVKMNCAAMPAGLLESDLFGHERGAFTGASSQRLGRFELADKSSLFLDEVGDMPLELQPKLLRVLQEQEFERLGSNKLIQTDVRLIAATNRDLKKMVADREFRSDLYYRLNVFPICLPPLRERPEDIPLLVKAFTAKIARRMGRNIDSIPAETLRTLSSMEWPGNVRELENVIERAVLLTRGNVLQLSLPEISLPDETLVAAEVAQNGEDEYQLIMRVLKETNGVVAGPKGAAQRLGLKRTTLLSRMKRLGIDKESLV

>WP_148576644.1 formate hydrogenlyase transcriptional activator [*Enterobacter* sp. Z1]

MPYTPMSDLGQQGLFDITRTLLQQPDLGALSDALTRLVRQSALADSAAIVLWHSASHRASYYSTRDNGKAFEYEDETYLAHGPVRRILSRPEALHCNFEEFSEAWPMLAQSQLYPPFGHYSLLPLAVEGHIFGGCEFIRHTDQPWSEAEYERLHTFTQIVAVVAEQIQSRVTNNVDYDLLSRERDNFRILVAITNAVLSRLDMDELVSEVSKEIHHYFKIDAISIALRGHRKGKLNIYSTHYLDEANPAHEQSEVDEAGTLSERVFKSKEILLLNLNEQDALAPYERMLFNTWGNKIQTLCLLPLMSGNTMLGVLKLAQCEEGVFTTANLKLLRQIAERISIALDNALAYQEIHRLKERLVDENLALTEQLNNVDSEFGEIIGRSDAMYSVLKQVEMVAQSDSTVLILGETGTGKELIARAIHNLSNRNSRRMVKMNCAAMPAGLLESDLFGHERGAFTGASSQRLGRFELADKSSLFLDEVGDMPLELQPKLLRVLQEQEFERLGSNKLIQTDVRLIAATNRDLKKMVADREFRSDLYYRLNVFPICLPPLRERPEDIPLLVKAFTAKIARRMGRNIDSIPAETLRTLSSMEWPGNVRELENVIERAVLLTRGNVLQLSLPEVSLPETPVTATDVAQEGEDEYQLIMRVLKETNGVVAGPKGAAQRLGLKRTTLLSRMKRLGIDKESLM

**Fig. S4. Reference amino acid sequences of FHLA in related species of *Enterobacter* for construction of the phylogenetic tree**

1. * Correspondence E-mail: jhtabszq@163.com; zhangqin@ahpu.edu.cn [↑](#footnote-ref-1)
